# SiRNA in MSC-derived exosomes silences CTGF gene for locomotor recovery in spinal cord injury rats

**DOI:** 10.1186/s13287-021-02401-x

**Published:** 2021-06-10

**Authors:** Wei Huang, Mingjia Qu, Lu Li, Tao Liu, Miaoman Lin, Xiaobing Yu

**Affiliations:** 1 Department of Orthopaedics, Affiliated Zhongshan Hospital of Dalian University, No. 6 Jiefang Street, Dalian, 116001 Liaoning Province China; 2 Department of Orthopaedics, Dongguan Tungwah Hospital, No.1 Dongcheng East Road, Dongcheng District, Dongguan, 523000 Guangdong Province China

**Keywords:** Spinal cord injury, Exosomes: Mesenchymal stem cells, RNA interference, Small interfering RNA

## Abstract

**Background:**

How to obtain a small interfering RNA (siRNA) vector has become a moot point in recent years. Exosomes (Exo) show advantages of long survival time in vivo, high transmission efficiency, and easy penetration across the blood-spinal cord barrier, renowned as excellent carriers of bioactive substances.

**Methods:**

We applied mesenchymal stem cell (MSC)-derived exosomes as the delivery of synthesized siRNA, which were extracted from rat bone marrow. We constructed exosomes-siRNA (Exo-siRNA) that could specifically silence CTGF gene in the injury sites by electroporation. During the administration, we injected Exo-siRNA into the tail vein of SCI rats,

**Results:**

In vivo and in vitro experiments showed that Exo-siRNA not only effectively inhibited the expressions of CTGF gene, but quenched inflammation, and thwarted neuronal apoptosis and reactive astrocytes and glial scar formation. Besides, it significantly upregulated several neurotrophic factors and anti-inflammatory factors, acting as a facilitator of locomotor recovery of rats with spinal cord injury (SCI).

**Conclusions:**

In conclusion, this study has combined the thoroughness of gene therapy and the excellent drug-loading characteristics of Exo for the precise treatment of SCI, which will shed new light on the drug-loading field of Exo.

## Background

Spinal cord injury (SCI) is one of the most serious complications of spinal fracture, which mostly results in severe sensory and motor dysfunction, even paraplegia [1, 2]. Recent studies have explained that the exosomes (Exo) secreted from mesenchymal stem cells (MSCs) may take the central role behind their potential therapeutic effect on SCI [3, 4]. Moreover, apart from Exo’s penetration across the blood-brain/spinal cord barrier depending on their small size, they also manifest a promising advantage of homing to injury sites of the central nervous system driven by inflammation [5, 6]. Their specific targeting provides the feasibility for drug delivery to the injured spinal cord segments.

Exosomes is nano-sized vesicles with the diameter of 30–200 nm secreted by various cells [7]. As an important means of cell-to-cell interaction, exosomes can enter target cells via receptors-mediated endocytosis, transmitting the messenger RNA (mRNA), small non-coding RNA, protein, and so forth to participate in the regulation of biological information [8, 9]. In the experiment of central nervous system injury, MSC-Exo have facilitated the growth of axons of cortical neurons. In stroke rats, MSC-Exo have been systemically administered to promote neurovascular remodeling and restore neurological function [10]. So MSC-Exo as a commensurate alternative therapy can be expected for stem cell therapy.

After SCI, connective tissue growth factor (CTGF) is involved in initial activation of glial cells in the injury sites [11]. Specifically, the overexpression of CTGF accelerates the proliferation of glial scars, thwarting axon migration and regeneration [12]. However, conditional deletion of CTGF significantly reverses the effect, reducing glial scar proliferation. Adenovirus- or lentiviral-mediated RNA interference (RNAi) is the mainstream method currently used as a RNA knockout technology, which can continuously silence specific genes by introducing target small interfering RNA (siRNA) into cells and continuously express it through virus infection [13]. Despite the strong infectivity and integration of viral vectors, these exogenous viruses can induce an immune response to transgenes [14]. Therefore, the preoccupation is to find a safer and more efficient siRNA delivery vector for clinical practice. As natural vesicles secreted by autologous cells, Exo have low immune rejection and can transmit biological genetic information through blood-brain barrier [15]. Therefore, they can be considered as ideal siRNA vectors.

In previous studies, Faruqu et al. succeeded in loading Atto655 labeled siRNA into HEK-293-derived exosomes by electroporation [16], and Farrukh Aqil et al. successfully introduced siRNA targeting Kirsten rat sarcoma viral oncogene homolog (KRAS) into milk-derived exosomes by the same method, showing good loading rate and silencing efficiency [17].

Herein, we hypothesized that siRNA encapsulated in MSC-Exo has a positive effect on the functional recovery after SCI. To address this hypothesis, we transfected the siRNA targeting CTGF gene into MSC-Exo by electroporation to construct Exo-siRNA, which was used to treat spinal cord injury in rat by tail vein injection. Relevant in vivo and in vitro experiments showed that Exo-siRNA not only effectively inhibited the expressions of CTGF gene and glial scar formation related proteins, such as GFAP, vimentin, fibronectin, and laminin in the injured spinal cord segments, but quenched inflammation and thwarted neuronal apoptosis. Besides, it significantly upregulated several neurotrophic factors and anti-inflammatory factors (e.g., TGF-*β*1), acting as a facilitator of axon regeneration. Thus, the motor function of SCI rats was significantly improved. In conclusion, this study has combined the thoroughness of gene therapy and the excellent drug-loading characteristics of Exo for the precise treatment of SCI, which will shed new light on the drug-loading field of Exo.

## Methods

### Preparation of MSCs and isolation of Exo

We isolated primary rat MSCs from 2-week-old male SD rats by whole bone marrow culture [18]. Simply put, the bone marrow of femur and tibia was washed with phosphate buffer saline (PBS) and centrifuged. The pellets were suspended in DMEM/F12 medium (Life Technologies, USA), then inoculated with 1 × 10^6^/ml cell concentration into a 25-cm^2^ flask containing 10% FBS (Life Technologies, USA) and 1% penicillin, and cultured in a 37 °C and 5% CO^2^ incubator.

As previously described by our laboratory, exosomes were isolated from the supernatant of MSCs by ultracentrifugation [19]. Briefly, the supernatant was centrifuged at 500×*g* for 5 min and at 2000×*g* for 30 min at 4 °C, mixed with the equivalent volume of pre-cooled 16% PEG600 (Jinhui, Shanghai, China). The mixed solution was centrifuged at 10,000×*g* (E5810R, Eppendorf, Germany) for 60 min at 4 °C to remove cells and debris. The resulting products were purified exosomes.

### Design and synthesis of Cy3-siRNA targeting CTGF gene

The CTGF gene sequence (sequence number ID 64032) in Genbank database was utilized to match the target sequence [13]. When designing siRNA, we did not analyze the non-coding regions at 5′ and 3′ ends to obtain sequences (Table 1). To provide the sequence of target gene fragment for further synthesis by Meixuan Biological Company (Shanghai, China), three pairs of target gene siRNAs corresponding to different regions were designed and labeled by fluorescent dye Cy3.

**Table 1:** Target sequence of three pairs of siRNA for *CTGF* gene

Name	Target sequence (5' to 3')
siRNA-1	CCGACTGGAAGACACATTT
siRNA-2	CCGGGTTACCAATGACAAT
siRNA-3	CCTGTCAAGTTTGAGCTTT

### Preliminarily analysis for the inhibition efficiency of RNAi

HEK293T cells (Saibao, Shanghai, China) were seeded in 6-well plates (6 × 10^5^ cells per well) and grown overnight at 37 °C with 5% CO_2_. SiRNA-1, 2, and 3 of 1 μg were cotransfected into HEK293T cells using Lipofectamine™ 3000 (Thermo, Shanghai, China). The HEK293T cells without transfection were used as a control. After cotransfection, the expression of CTGF gene in HEK293T cells was detected at 72 h by fluorescence microscope to preliminarily analyze the inhibition efficiency of RNAi. At the same time, the total RNA of transfected cells was extracted and CTGF-related mRNAs were analyzed by Real-Time (RT)-PCR.

### The loading efficiency of siRNA

As was mentioned earlier [16], we intended to load siRNA into the PKH67-Exo by electroporation using the EC630 electroporation system (BTX, USA). PKH67-Exo was diluted to 0.5 mg/mL in siRNA electroporation buffer (BTX, USA). Target gene Cy3-siRNA and fluorescently labeled control siRNA were added at a final concentration of 100 nM. The mixtures were transferred into ice-cold 0.4-cm cuvettes and electroporated under 8 different conditions, involving changes in voltage, resistance, and capacitance (Table 2) with a pulse time of 10–15 ms. Electrical perforation was performed once in each condition according to the reference system [17]. When electroporation was accomplished, exosomes were extracted from electroporation buffer and siRNA extraction, so double fluorescent-labeled Exo-siRNA were obtained for reversing transcription and qPCR .

**Table 2:** Grouping according to different electroporation conditions

Conditions/groups	1	2	3	4	5	6	7	8
Voltage (v)	400	400	400	400	400		400	400
Resistance (Ω)	50	50	50	50	25		150	150
Capacitor (μF)	500	200	300	400	500		500	500
Exosome volume (μL)	7	7	7	7	7	7	21	21
siRNA volume (μL)	3.75	3.75	3.75	3.75	3.75	3.75	10	20

SiRNA concentration, 20μM; exosome concentration, 2μg/uL

System, 3.75 μL, 20 μM, siRNA+ 7 μg exosome; the rest volume was 150 μL supplemented with buffer

Group 6 is the control group without electroporation

### Quantitative determination of siRNA-loading capacity in exosomes

#### Loading capacity

To determine siRNA-loading capacity, the 32P-labeled siRNA was adopted. Radioactivity in the pellet and supernatant was determined by a known volume to a PEI-cellulose layer and quantitated by Packard InstantImager. The loading rate was calculated by the following formula: siRNA loading = (CPM in pellet/CPM in supernatant) ×100. The siRNA-loading capacity was calculated by the aforesaid formula.

#### SiRNA stability test

Exo-siRNA were incubated with various concentrations of RNase A (0.01 μg/mL, Sigma, USA). After incubation at 37 °C for 45 min, naked siRNA was used as control. Quantitative analysis of undergraded siRNA was performed by RT-PCR.

### Co-culture of Exo-siRNA with astrocytes, dorsal root ganglions, and macrophages in SCI environment

#### Co-culture with astrocytes

Rat astrocytes (ATCC, CAS cell bank) were inoculated on 12-well plate, with 5 × 10^4^ cell/well. When astrocytes fused to about 90%, the SCI environment was simulated by 100 μL Lipopolysaccharide (LPS, Jinhui, China) and hypoxia (2% O_2_) for 24 h. Cell grouping is as follows: siRNA-Lipo group (*n = 3*) was incubated by 50 nM siRNA and 2 μL lipo3000 (Thermo, L3000015); 50 nM siRNA (without lipo3000) were added as Untransfected group (*n = 3*); Exo-siRNA group contained 50 μL Exo-siRNA (*n = 3*). RNAs were extracted 48 h after incubation for further quantitative RT-PCR analysis. Proteins were also extracted for Western blotting.

#### Co-culture with dorsal root ganglions

Rat spinal dorsal root ganglions (DRGs, ATCC, CAS cell bank) were sampled. Cell grouping as follows: SCI group (*n = 3*) was treated with 50 μL phosphate buffer saline (PBS); siRNA group (*n = 3*) was incubated by 2 μL lipo3000 and 50 nM siRNA; Exo group (*n = 3*) was added with 50 μL Exo; Exo-siRNA group (*n = 3*) contained 50 μL Exo-siRNA.It was noted that cells of the Control group (*n = 3*)were not treated with LPS/hypoxia. After the 24-h administration, DRGs neurons were washed with PBS twice and stained in situ. Each 12-well plate was rearranged with 200 μL Calcein-AM/PI double staining reagent (Saibaikang, HR0444, China). Yellow/green fluorescence indicated living cells, and red fluorescence marked dead cells. Cell viability was detected by CCK-8 solution (Abbkine, KТC011001).

#### Co-culture with macrophages

Rats NR8383 macrophages (ATCC, CAS cell bank) were obtained. Cell culture, seed plate, grouping, and administration were the same as those of DRGs, no Control group was set. After 48-h treatment, RNAs were extracted for RT-PCR analysis and an ELISA assay was adopted to detect expressions of various inflammatory factors using the following kits: rat TNF-*α*, IL-6, IL-8, and IL-10 ELISA kit (Cusabio, China).

### Establishment, grouping, and tail vein administration of SCI rat models

The animal experiments in this study were reviewed and approved by the ethics committee of Affiliated Zhongshan Hospital of Dalian University. One hundred SD female rats (200–250 g, 10 weeks old) were randomly divided into 5 groups: SCI group (PBS treatment), Sham group (PBS treatment), siRNA group (siRNA treatment), Exo group (pure exosomes treatment), and Exo-siRNA group (Exo-siRNA treatment) with 20 rats in each group. Rats were anaesthetized with sodium pentobarbital (Sigma, St. Louis Mo, USA). An incision of 2 cm was made at T10 level to expose skeletal muscles of spinal cord in sterile environment. Except for the Sham group, receiving a sham operation, rats of the rest groups were hit on the T10 segment using a spinal cord impactor (PSI, IH-0400, USA), with metal rod weight of 30 g and height of 50 mm. After that, bilateral hind limbs were involuntarily twitching and tails swinging in all animals, indicating that the injury corresponded to the standard of a SCI model. After the suture of muscle layer and skin, rats were allowed to recover in heat preservation chambers. Postoperative nursing of SCI rats mainly encompassed daily artificial bladder emptying and restoration of bladder reflex activity. Tail vein administration was performed every 24 h for 5 consecutive days, and the dosage was as follows: SCI group and Sham group were administered with 100 μL PBS; while siRNA group with 100nM siRNA and 4 μL lipo3000 in 100 μL PBS, Exo group with 100 μL Exo solution and Exo-siRNA group with 100 μL Exo-siRNA solution.

### Toxicity test of Exo-siRNA on normal health rat liver and lung

#### H-E staining of lung tissue in normal health rats

According to H-E staining kit (Wanlei Biology, China), after the normal rats were given Exo-siRNA for 24 h, the nucleus was blue and the cytoplasm was pink.

#### Detection of lactate dehydrogenase (LDH), alanine aminotransferase (ALT), and superoxide dismutase (SOD) in normal health rat liver

After 24 h of Exo-siRNA treatment in normal healthy rats, the liver of the rats was taken. The activity of LDH was determined by micro enzyme labeling method. 10μL rat liver homogenate was taken and the cells were lysed by PBS. The cell culture medium could be used to determine the activity of LDH directly by kit (WanleiBio, China). The activity of ALT was measured by visible light colorimetry. The activity of SOD was determined by visible spectrophotometry.

### Quantitative qRT-PCR

Next, we extracted total RNA from various cells and spinal cord tissues. SiRNAs from Exo or cells were extracted by a miRNA extraction kit (Solarbio, China). Target mRNA or siRNA was used as template, and the total RNA reverse-transcribed into cDNA using a reverse transcription kit (Solarbio, China). The volume of the whole reaction system was 15 μL. *Gapdh* was adopted as an internal reference gene for relative quantification by the 2^−∆∆Ct^ method. All primer sequences of mouse gene used in qRT-PCR are shown in the Table 3.

**Table 3:** The primer sequences of mouse gene used in qRT-PCR

Mouse gene	Primer sequence
IL-6	F:5′-GGTGCCCTGCCAGTATTCTC-3' R:5′-GGCTCCCAACACAGGATGA-3'
TNF-α	F:5′-AAGCCTGTAGCCCACGTCGTA-3' R:5′-GGCACCACTAGTTGGTTGTCTTTG-3'
Arg-1	F:5′-CTCCAAGCCAAAGTCCTTAGAG-3' R:5′-GGAGCTGTCATTAGGGACATCA-3'
CD206	F:5′-AAACACAGACTGACCCTTCCC-3' R:5′-GTTAGTGTACCGCACCCTCC-3'
GAPDH	F:5′-GTGCTGAGTATGTCGTGGAGTCT-3′ R: 5′-GTGGAAGAATGGGAGTTGCTGT-3′

### Western blotting (WB)

Total protein of exosomes, cells, or spinal cord tissues was extracted using RIPA buffer and protein concentration was evaluated with a BCA assay according to the manufacturer’s protocol. Perform WB experiments according to the conventional process; all primary antibodies included CD9 (rabbit), CD63 (rabbit), GAPDH (rabbit), β-actin (rabbit), CTGF (rabbit), GFAP (rabbit), vimentin (rabbit), fibronectin (rabbit), laminin (rabbit), BDNF (rabbit), and TGF-*β*1(rabbit, Abcam, UK).

### Masson staining

After 28 days, the spinal cords of all rats were harvested and sectioned (2 mm) in cross section. After dewaxing, the samples were washed with distilled water three times. Then, they were stained with nuclear dye for 2 min, and destained with 1% hydrochloric acid alcohol for 1 s, blue in running tap prior to cytoplasmatic staining for 30–60 s. Finally, the slides were dehydrated and the tissue was mounted with a coverslip. The relative quantification of glial scar area was calculated by Image J software (V1.8.0.112).

### Behavioral assessment of the locomotor function

#### Neurophysiological experiment

On the 48th day after injury, sodium pentobarbital (Sigma, USA) was used to anesthetize animals in each group. Transcranial electrical stimulation was applied with pulse, and SEN-7301 constant current isolation stimulator was used for 1 ms at 9 mv (Digitizer). Use two transdermal stimulants to place 30 g stainless steel stimulation electrodes (Photoelectric, Japan). Motor evoked potentials (MEPs) were recorded with two hindlimb ring electrodes.

#### Quantification of urine spot areas

On the 48th day after administration, all animals were separately placed in a cage of 16 × 30 cm, and the entire floor area was covered with filter paper to measure the urination pattern [20]. One hour later, the filter paper was collected, and the abnormal image analysis software (Molecular equipment) was used to scan and calibrate each urine spot, and the urine spot area was measured.

#### Thermal sensitivity

We also used paw withdraw thermal latency (PWTL) to judge the thermal sensitivity of rats. A radiant heat source (model 390G, IITC Company, USA) was placed in the middle of the plantar surface of the hind paw of the rat. After the hind paw moved or 20 s later, the heat stimulator would automatically turn off to avoid tissue damage. The effects of 50% radiant heat intensity and 6–8-s thermal arrest latency on normal pain threshold were detected. The pain threshold time of rats under free condition was measured automatically.

#### Basso, Beattie, and Bresnahan (BBB) scale

Rat locomotor recovery was estimated by the BBB locomotor rating scale, ranging from 0 (no motor activity) to 21 points (normal locomotion). The treatment assessed the locomotor activity with corresponding scores at 1, 7, 14, 21, 28, 35, 42, and 48 days after injuring.

### Statistical analysis

Statistical analyses were performed using MATLAB/GraphPad Prism software. Student *t* test was utilized for two group comparison. One-way ANOVA was adopted to compare the differences between groups while multiple comparisons were performed by Tukey’s post hoc test (GraphPad Prism 8.0). *p* <0.05 indicated statistical significance. Significance levels were represented as *p < 0.05 and **p < 0.01.

## Results

### Characterization of MCSs-Exo and Exo-siRNAs

**Fig. 1:**
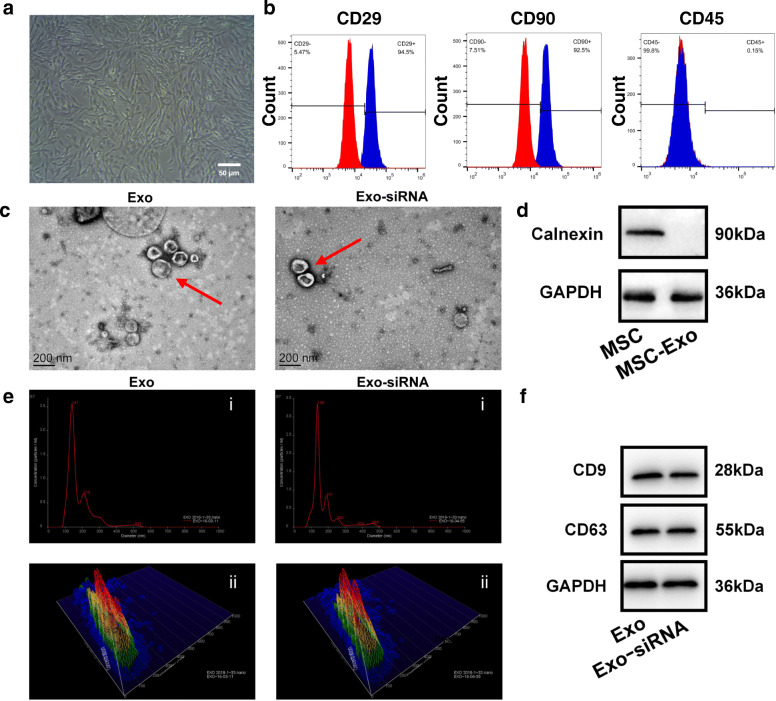
Characterization of MSCs, MSC-Exo, and Exo-siRNA. **a** Morphology of MSCs under microscope. Bar = 50 μm. **b** The surface antigens CD29, CD90, and CD45 of MSCs were identified by flow cytometry. **c** TEM revealed the typical exosome structure of Exo and Exo-siRNA. The red arrow points to the Exo and Exo-siRNA. Bar = 200 nm. **d** The rat bone marrow MSCs marker Calnexin were analyzed by WB. **e** NTA analysis of Exo and Exo-siRNA showed the incorporation of siRNA did not affect the diameter of Exo. **f** The MSC-Exo marker proteins CD9 and CD63 were analyzed by WB

Firstly, We have successfully extracted rat bone marrow MSCs. When the cells were subcultured, the morphology of MSCs was single, spindle, or flat, arranged in the shape of vortex and radiation (Fig. 1a). The results of flow cytometry also showed that MSCs highly expressed CD29 (94.5%) and CD90 (92.5%) and lowly expressed CD45 (0.15%), which were consistent with the characteristics of rat bone marrow MSCs (Fig. 1b). After extraction of exosomes, we carried out a series of exosomes identification steps. Firstly, transmission electron microscopy (TEM) revealed that the typical saucer-like morphology with the diameter of 30–200 nm was found in both Exo and Exo-siRNA (Fig. 1c). Next, we made calnexin WB of MSCs and MSC-Exo. The results showed that the expression of calnexin of MSCs was significantly higher than that MSC-Exo, which proved that calnexin was a unique marker of cells (Fig. 1d). Simultaneously, characterization of Exo and the corresponding Exo-siRNA were preliminarily compared. Nanoparticle tracking analysis (NTA) also showed that most Exo and Exo-siRNA were around 150 nm in diameter with little difference, indicating that the incorporation of siRNA did not affect the shape and diameter of Exo (Fig. 1e). Finally, the results of WB suggested that the expressions of exosome markers, CD9 and CD63, were impervious to siRNA loading (Fig. 1f).

### The MSC-Exo presented high loading rate of siRNA with high stability

**Fig. 2:**
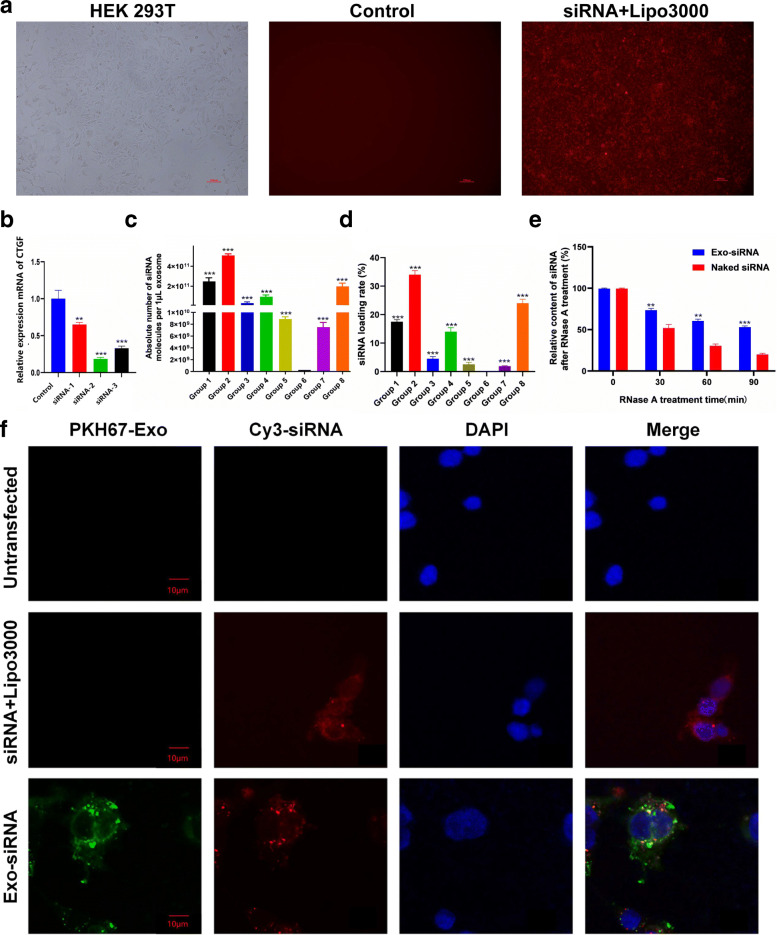
Preliminarily analysis of silencing efficiency of three pairs of target siRNA. **a** Cy3-siRNA was transfected into HEK293T cells by Lipo3000, and the control group was not transfected. Bar = 100 μm. **b** The expressions of CTGF-related mRNAs at 72 h after siRNA transfection were presented. *n =* 3, data are represented as *mean ± SD,* *p < 0.05, **p < 0.01 vs control. **c** The absolute number of siRNA molecules in Exo under different electroporation conditions was exhibited. *n =* 3, data are represented as *mean ± SD*, *p < 0.05, **p < 0.01 vs group 6. **d** The loading rates of siRNA in Exo under different electroporation conditions were presented. *n =* 3, data are represented as *mean ± SD*, *p < 0.05, **p < 0.01 vs group 6. **e** Relative contents of naked siRNA and Exo-siRNA after RNase treatment were showed. *n =* 3, data are represented as *mean ± SD,* *p < 0.05, **p < 0.01 vs naked siRNA. **f** Fluorescence patterns of LPS/hypoxia-treated astrocytes after co-culture with PKH67 and Cy3 double-labeled Exo-siRNA for 72 h were assessed. Bar =10 μm

In the following experiment, we transfected HEK293T cells with these siRNAs targeting CTGF gene by Lipofectamine™ 3000 (Fig. 2a), and the RT-PCR results showed that all the siRNAs can inhibit the expression of CTGF gene to a certain extent, ranging from 18.85 ± 7.75 to 65.52 ± 7.52%. As a result, siRNA-2 presented stronger inhibitory effect than the other two siRNA (Fig. 2b), which was preferable to be loaded on the exosome. So we loaded siRNA-2 into MSC-Exo by electroporation at 8 different conditions. After electroporation, the absolute quantitative RT-PCR results showed that exosomes of the 8 groups were loaded with a certain amount of siRNAs. The loading efficiency of siRNA was the highest in group 2, the count of siRNA nanoparticles per microgram of exosomes was about 5.08×10^11^/μg (Fig. 2c), indicating that its electroporation system of group 2 was optimum.

Subsequently, we measured the loading rate of siRNA in exosomes and the results showed that the loading rate of siRNA was consistent with the corresponding RT-PCR results. Group 2 achieved the highest siRNA-loading rate/electroporation efficiency, of about 33.24% (Fig. 2d). So the optimal parameters for electroporation system are as follows: voltage, 400 v; resistance, 50 Ω; capacitor, 200 μF; exosome volume, 7 μL; and siRNA volume, 3.75 μL.

In order to test the stability/anti-degradation ability of Exo-siRNA, we cultured the Exo-siRNA with RNase A (simulating body fluid environment) and naked siRNA as the control group for 45 min. As a result, the content of siRNA in Exo-siRNA was significantly higher than that of naked siRNA by RT-PCR. After 45-min RNase A treatment, 75.1% siRNA residues remained in the Exo-siRNA group, compared with less than 50% in the naked siRNA group. This indicated that exosomes as natural carriers of siRNA showed favorable encapsulation ability and effectively prevented the degradation of siRNA in body fluids (Fig. 2e).

### In vitro study of Exo-siRNA as a treatment for SCI

#### Exo-siRNA effectively inhibited the expression of CTGF gene in astrocytes and stimulated the secretion of neurotrophic factors and anti-inflammatory factors in the impaired spinal cord

**Fig. 3:**
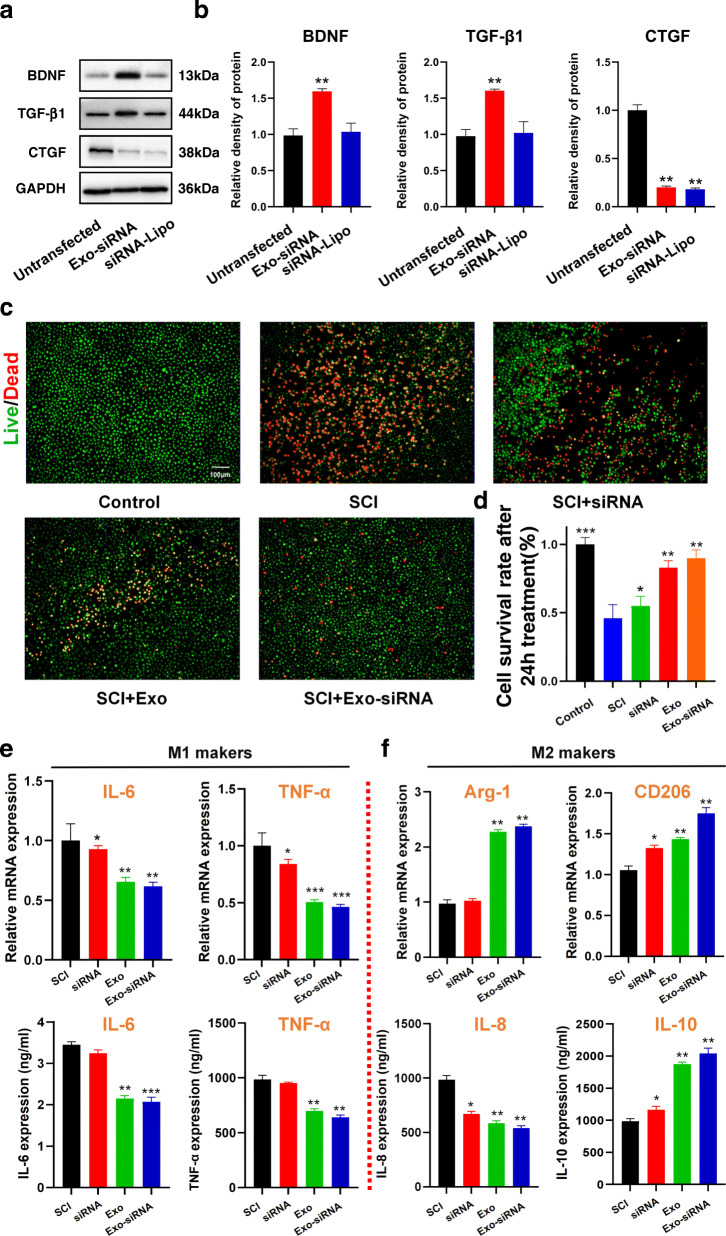
Exo-siRNA could effectively inhibit the expression of CTGF gene in astrocytes and upregulate the secretion of neurotrophic factors and anti-inflammatory factors in the impaired spinal cord segments. **a** The WB of CTGF*,* BDNF, and TGF-*β*1 after Exo-siRNA co-culture for 72 h was determined, *n =* 3, data are represented as *mean ± SD,* *p < 0.05, **p < 0.01 vs control. **b** The proteins relative expressions of CTGF, BDNF, and TGF-*β*1 at 72 h after Exo-siRNA treatment were detected. *n =* 3, data are represented as *mean ± SD,* *p < 0.05, **p < 0.01 vs control. **c** Living and death staining patterns of LPS/hypoxia-treated DRGs were analyzed at 24 h after co-culture with Exo-siRNA, siRNA, Exo, and PBS, respectively. Living and dead cells were stained with calcein-AM (green) and propidium iodide (red), while the control group did not receive any treatment. Scale bar =100 μm. **d** Cell survival rate of LPS/hypoxia-treated DRGs was calculated by CCK-8 at 24 h after co-culture with Exo-siRNA, siRNA, Exo, and PBS. *n =* 3, data are represented as mean ± SD, *p < 0.05, **p < 0.01 vs LPS/hypoxia. **e**, **f** qRT-PCR analysis was performed for mRNA expressions and ELISA analysis for secretion of **e** M1-related pro-inflammatory cytokines and **f** markers of M2 macrophage phenotypes in LPS/hypoxia-treated NR8383 macrophages at 48 h after Exo-siRNA, siRNA, Exo, and PBS administrations. *n =* 3, data are represented as mean ± SD, *p < 0.05, **p < 0.01 vs LPS/hypoxia

Here, we investigated the underlying effect of Exo-siRNAs on SCI. Rat astrocytes were treated with LPS for simulating the environment of SCI and then cultured under hypoxia (2% O_2_) for 24 h. Subsequently, Exo-siRNA was co-cultured with rat astrocytes. Conventional transfection using Lipo3000 transfection reagent was used as the control group. After 24-h administrations, confocal fluorescence microscopy revealed that the cells treated with Exo-siRNA were double-positive for green fluorescence (Exo) and red fluorescence (siRNAs) in Fig. 2f. WB uncovered that both Exo-siRNA group and the control group significantly downregulated the expression of CTGF gene, with the downregulatory rates of 21% and 19%, respectively (Fig. 3a, b). This indicated that Exo were competent in delivering siRNA for interfering target genes. Besides, WB results showed that Exo-siRNA facilitated secretions of neurotrophic factors (e.g., BDNF) and anti-inflammatory factors (e.g., TGF-β1) by astrocytes, which was undoubtedly of therapeutic significance of SCI. The levels of BDNF and TGF-β1in the Exo-siRNA group were 72.2% and 61.2% higher than those in the untransfected group, respectively (Fig. 3a, b).

#### Exo-siRNA thwarted neuronal apoptosis in the impaired spinal cord segments

A previous study reported inhibitory effect of MSC-Exo, acting as neural protectors, on neuronal apoptosis and inflammatory factors [21]. In this study, Exo-siRNA was co-cultured with LPS and DRGs of hypoxia-treated rats for 24 h. After the 24-h administrations, cell viability was assessed by living and death staining (Fig. 3c) and quantitative analysis of Cell Counting Kit-8 (CCK-8). In Fig. 3d, it was found that the survival rate of DRGs in SCI group was significantly reduced by less than 50%. Exo and Exo-siRNA treatment groups showed higher survival rates of DRGs than SCI group, which were close to 90.0%. There was nonsignificant difference between the two groups, indicating that the anti-apoptotic effect of Exo-siRNA mainly attributed to Exo.

#### Exo-siRNA propelled the balance of macrophages towards M2 in SCI environment and quenched inflammation

According to previous researches, MSC-Exo can induce macrophage polarization and inhibit inflammation in SCI [22]. Here, we explored the promoting effect of Exo-siRNA on macrophage polarization. NR8383 cells derived from rat alveolar macrophages were cultured with LPS/hypoxia for 24 h and then transformed into M1 state. After 48-h incubation of siRNA, MSC-Exo, and Exo-siRNA, the qRT-PCR and ELISA results showed that the expressions of M1-related pro-inflammatory cytokines, TNF-*α* and IL-6, significantly dropped in Exo or Exo-siRNA groups with nonsignificant differences between the two groups (Fig. 3e). Administrations of Exo and Exo-siRNA significantly boosted mRNA expressions of M2 macrophage markers, Arg-1 and CD206, without significant difference between the two groups. The expressions of anti-inflammatory factors, IL-8 and IL-10, in M2 macrophages were remarkably elevated in Exo and Exo-siRNA treatment groups (Fig. 3f). However, these changes were not evident in siRNA group, which indicated that siRNA may not direct regulatory effect on inflammatory factors.

### In vivo study of Exo-siRNA in the treatment of SCI

#### Exo-siRNA passed through the blood/spinal cord barrier and accumulated at the impaired sites of SCI

**Fig. 4:**
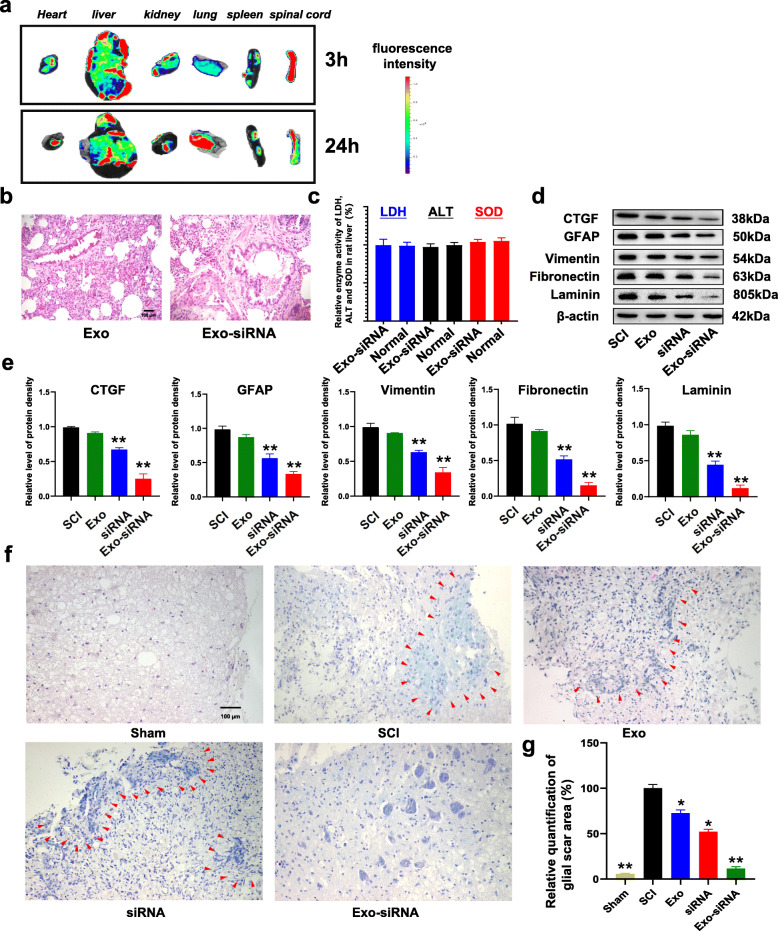
Therapeutic effects of Exo-siRNA on SCI rats. Rats were classified into SCI group, Sham group, Exo group, and Exo-siRNA group, *n* = 6. **a** Fluorescence imaging of organs of Exo-siRNA group at 3 h, 24 h, and 48 h after intravenous injection was illustrated. **b** The results of H-E staining in the lungs of normal healthy rats in the Exo and Exo-siRNA group. Bars = 100 μm. **c** The relative enzyme activity of LDH, ALT, and SOD in rat liver. **d** Expressions of CTGF, GFAP, vimentin, fibronectin, and laminin were assessed by WB, *n =* 3, data are represented as *mean ± SD,* *p < 0.05, **p < 0.01 vs SCI group. **e** The relative level of CTGF, GFAP, vimentin, fibronectin, and laminin protein density. *n =* 3, data are represented as *mean ± SD,* *p < 0.05, **p < 0.01 vs SCI group. **f** Transverse sections of core lesion tissues in each group were stained by Masson’s trichrome method. Fibrotic tissue area was indicated by red arrows. Bars = 100 μm. **g** Relative quantification of glial scar area (%), *n=*3, data are represented as *mean ± SD,* *p < 0.05, **p < 0.01 vs SCI group

We further investigated the therapeutic effect of Exo-siRNA on SCI rats. Exo and Exo-siRNA were administered to SCI rats by intravenous tail vein injection, and its targeting to spinal cord segments was traced by living animal fluorescence imaging system. The results show that a majority of Exo and Exo-siRNA accumulated in the liver and bilateral lungs (Fig. 4a). It was found that Exo and Exo-siRNA presented relatively high fluorescence intensity in the liver based on the 3-h imaging. Exo and Exo-siRNA were mainly absorbed by mononuclear phagocytes in the liver after systemic injection, and there was fluorescence in the spinal cord, indicating chemotactic activity for Exo and Exo-siRNA in the injured spinal cord segments. Twenty-four hours after injection, Exo and Exo-siRNA were absorbed by various nerve cells in the liver and injured spinal cord.

#### Detection of drug toxicity of Exo-siRNA to liver and lung

In order to detect the toxic effect of Exo-siRNA on liver and lung, we did some liver toxicity tests and H-E staining of lung tissue after giving Exo-siRNA to normal health rats. According to the results of lung pathology, the alveolar structure and lung cell morphology of rats were normal, and no obvious damage was found in lung tissue (Fig. 4b).

LDH and ALT were a stable protein, once the cell membrane is damaged, LDH and ALT were released to the outside of cells [23]. By detecting the LDH and ALT activity in the cell culture supernatant, the degree of cell damage can be determined; in addition, SOD also plays an important role in the balance of oxidation and antioxidation. It can scavenge superoxide anion free radicals (O2−) and protect cells from damage [24]. So we use these three indicators to measure the toxicity of Exo-siRNA to the liver. The results showed that the enzyme activity of LDH, ALT, and SOD in the liver of Exo-siRNA group were similar to those of normal rats, which shows that Exo-siRNA has no toxic effect on the liver of rats, and is a relatively safe nano drug (Fig. 4c).

#### Exo-siRNA not only significantly reduced the expression of CTGF gene, but also inhibited the expression of GFAP, vimentin, fibronectin, and laminin, thereby inhibiting the formation of glial scar

To verify the silencing effect of Exo-siRNA in vivo, SCI rats received intravenous injection of Exo and Exo-siRNA for 5 consecutive days from the date of modeling, and the rats in sham operation group and control group were administered with the same volume of saline. At the 4th week, the damaged segments were sampled, and protein was extracted for WB analyses. Compared with SCI rats, the proteins levels of CTGF and its downstream proteins in Exo-siRNA or siRNA rats were significantly decreased (Fig. 4d, e). It has been proved that CTGF-siRNA can downregulate the expressions of proteins related to glial scar formation, including GFAP, vimentin, fibronectin, and laminin [13]. Therefore, we used WB to measure the levels of these four proteins. As a result, the four proteins were downregulated in both siRNA and Exo-siRNA group compared with SCI group. The level of GFAP protein decreased to 31.7% and that of vimentin was 35.6%, fibronectin 15.2%, and laminin 10.5% in Exo-siRNA group and GFAP 55.8%, vimentin 63.5%, fibronectin 51.1%, and laminin 44.9% in siRNA group (Fig. 4d, e). Moreover, the injured spinal cord tissue of each group was collected at the 4th week. The formation of glial scar was measured by Masson staining (Fig. 4f). Compared with group SCI group, the area of glial scar in Exo-siRNA group was downregulated by 88.2% and the area of glial scar in siRNA group was 48.4% lower than that in SCI group (Fig. 4g). Interestingly, the area of glial scar in Exo group was also reduced by 26.5% compared with SCI group, but the downregulation rate was lower than that in Exo-siRNA group or siRNA group (Fig. 4g). These results indicate that CTGF-siRNA is the main factor in inhibiting the formation of glial scar.

#### Exo-siRNA promotes the recovery of neuroelectrophysiology in SCI rats

**Fig. 5:**
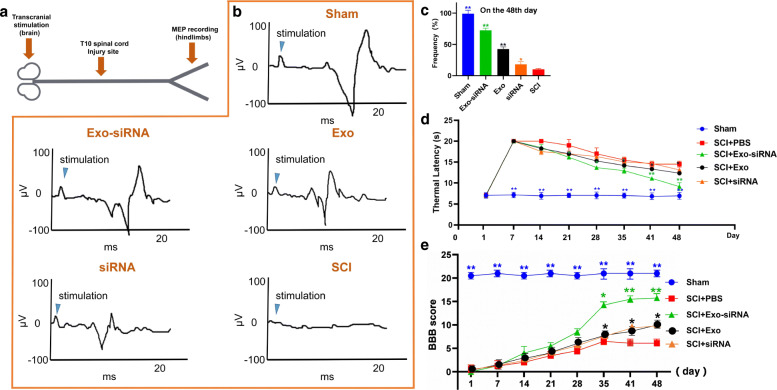
**a** The motor cortex of the brain was stimulated by transcranial electrical stimulation, and then MEPs of the hind limbs were recorded *(n=3)*. **b** MEPs of rats in each group after 48 days of treatment (the mean values recorded 3 times were collected into a line chart, the blue arrow marks the stimulus artifact). **c** The relative quantification of urine spot area on the 48th day after administration. *n =* 6, data are represented as *mean ± SD,* *p < 0.05, **p < 0.01 vs Sham group. **d** The paw withdraw thermal latency of rats in SCI group, Sham group, Exo group, siRNA group, and Exo-siRNA group. *n =* 3, data are represented as *mean ± SD,* *p < 0.05, **p < 0.01 vs SCI group. **e** Recovery of motor dysfunction of rats in SCI group, Sham group, Exo group and Exo-siRNA group were analyzed by BBB score. *n = 6*, data are represented as *mean ± SD,* *p < 0.05, **p < 0.01 vs SCI group

On the 48th day after injury, we used MEPs to measure the electrophysiological recovery of the rats (Fig. 5a). The results showed that the MEPs of rats in the Exo-siRNA group presented obvious depolarization and the average MEP amplitude (189±4μV) were significantly larger than the Exo, siRNA and SCI groups rats, while the neural recovery in the SCI group rats were poor and the intracranial stimulation hardly caused obvious depolarization (Fig. 5b).

#### Exo-siRNA ameliorated behavioral dysfunction of SCI rats

In addition, the results of urine spot area also showed that the urination behavior of rats in each group was also different. In Fig. 5c, due to the uncoordinated urination, the area of urine spots was relatively small. On the contrary, Exo-siRNA treated rats showed free and active urination, and the relative area of urine spots was larger, while the area of urine spots in Exo group and siRNA group was larger than that in SCI group, but still less than that in Exo-siRNA group. These results suggested that Exo-siRNA can improve the urination function of these animals by restoring neurogenic bladder dysfunction.

In terms of thermal sensitivity, the results of PWTL showed that the rats in all groups showed almost the same PWTL on the first day (before injury), which was about 7 s (Fig. 5d). One week after injury, except Sham group, the PWTL of all groups reached 20 s (Fig. 5d), because this was the acute stage of spinal cord injury, and the sensory nerve was basically paralyzed. As time went on, the PWTL of Exo-siRNA group decreased gradually, the decrease was about 9 s (Fig. 5d) on the 48th day and the rats in SCI group also had some self-recovery function, but the decrease was not as obvious as that other treatment groups, which was only 14.5 s on the 48th day (Fig. 5d). Therefore, Exo-siRNA has a certain effect on the recovery of sensory nerve.

Finally, we used BBB scores to evaluate the recovery of motor function of rats in each group and the results show that the BBB scores of all groups increased gradually with time, which indicated that rats had a certain ability of self-recovery. On the 48th day, Exo and Exo-siRNA groups exhibited significant functional recovery of locomotor activity compared with SCI group. The average score of rats in Exo-siRNA group was 15.4 + 1.5 points, and that in Exo group was 10.8 + 0.5 points (Fig. 5e).

## Discussion

The present study demonstrated that MSC exosomes loaded with CTGF-siRNA effectively inhibited the expressions of CTGF mRNA and glial scarring-related proteins, curbed inflammatory responses and neuronal apoptosis, upregulated the levels of BDNF and anti-inflammatory cytokines, so that the motor function in SCI rats was remarkably ameliorated.

Combining results of Table 2 and Fig. 2c, it was found that the lower the capacitance, the more siRNA molecules were loaded into Exo under the same voltage and resistance conditions in group 1 to 4. In comparison with group 7 and 8, group 8 presented fewer number of siRNA molecules loaded into Exo, with more siRNAs remaining in the reaction solution, than group 7 under the same voltage, resistance, and capacitance conditions. This indicated that the same electroporation condition often corresponded to an optimum concentration of siRNAs. Group 8 was the control group without electric shock, but a certain amount of siRNA could still be detected. This can be explained by the non-specific binding of siRNA to Exo.

Besides, Exo-siRNA also stimulated the expression of BDNF, especially in the central nervous system to support sensory neurons in the neural crest. Indeed, BDNF is essential in neuronal survival, differentiation, and the regulation of synaptic plasticity [25]. The re-expression of BDNF can be stimulated by itself, thereby inducing neuroprotection with favorable prognosis after SCI. In the present study, we investigated whether Exo-siRNA could upregulate the expression of BDNF in SCI rats and whether it could reduce secondary neural tissue damage and functional impairments of the spinal cord. Exogenous administration with BDNF can slow neural atrophy in the corticospinal tract, the rubrospinal tract, and the whole spinal cord. All these indicate one of the most significant functions of BDNF of minimizing axonal injury in the damaged spinal cord. On top of that, other significant functions encompass promoting axonal growth and regeneration. In the current study, we found that Exo-siRNAs upregulated BDNF expressions and increased the distribution of white matter areas and the number of oligodendrocytes after SCI. Taken together, these findings suggest that the Exo-siRNA-induced increases in spinal BDNF levels exert neuroprotection following SCI.

As most Exo and Exo-siRNA enriching in the liver and lung, they may bring toxic effects on the two organs. Among some studies about the tail vein administration of Exo in small animals, most of them tracked the distribution and enrichment of exosomes in major organs using in vivo fluorescence imaging. We also separately detected the drug toxicity in the liver and lung and found that Exo-siRNAs had no significant toxic and side effects on the liver and lung. Besides, it is also reported that exosomes have satisfactory immunogenicity to target organs without harmful effects; and according to evidences from cell experiments, a cell-killing effect of exosomes has not been found [26]. Thus, we speculate that exosomes have nonsignificant toxicity to the liver, lung and other organs.

Glial scar is a natural barrier for axonal regeneration, and CTGF plays an important role in the regulation of glial scar [27]. Previous studies have shown that CTGF can stimulate glial scar formation by mediating downstream related proteins, such as vimentin, fibronectin, and laminin [12, 13]. Vimentin is the intermediate filament of protein family, mainly in human glial cells, once the central nervous system is injured, vimentin will be significantly expressed [28, 29]. In particular, vimentin can play an important role in the formation of glial scar tissue. Fibronectin and laminin are the types of extracellular matrix collagen. After SCI, the damaged fibroblasts invaded the damaged area and secreted a large amount of extracellular matrix, including type IV collagen, fibronectin, and laminin, which are the main components of glial scar. The formation of glial scar can stimulate the production of chondroitin sulfate proteoglycans, which can activate RhoA signal, leading to growth cone collapse and inhibiting axon regeneration [12, 27, 30]. Our study used siRNA against CTGF gene to silence the expression of CTGF gene in vivo. The results also confirmed that siRNA can effectively reduce the expression of CTGF in the injured area, thus reducing the generation of glial scar, clearing the way for the regeneration of nerve axons. So, the locomotor function has been a significant recovery.

## Conclusions

SiRNA-induced CTGF knockdown attenuates injury-stimulated astrogliosis and the resultant scar formation, as well as GFAP production, thereby facilitating the recovery of locomotor function following SCI.

## Data Availability

The data that support the findings of this study are available from the corresponding author upon reasonable request.

## Abbreviations

Exo: Exosomes
siRNA: Small interfering RNA
MSCs: Mesenchymal stem cells
SCI: Spinal cord injury
mRNA: Messenger RNA
CTGF: Connective tissue growth factor
LPS: Lipopolysaccharide
PBS: Phosphate buffer saline
KRAS: Kirsten rat sarcoma viral oncogene homolog
DRGs: Rat spinal dorsal root ganglions
LDH: Detection of Lactate dehydrogenase
ALT: Alanine aminotransferase
SOD: Superoxide dismutase
WB: Western blotting
PWTL: Paw withdraw thermal latency
GFAP: Glial fibrillary acidic protein
NF-H: Neurofilament-H
TEM: Transmission electron microscopy
NTA: Nanoparticle tracking analysis
